# Translating phage therapy into the clinic: Recent accomplishments but continuing challenges

**DOI:** 10.1371/journal.pbio.3002119

**Published:** 2023-05-23

**Authors:** Aleksandra Petrovic Fabijan, Jonathan Iredell, Katarzyna Danis-Wlodarczyk, Razieh Kebriaei, Stephen T. Abedon

**Affiliations:** 1 Centre for Infectious Diseases and Microbiology, Westmead Institute for Medical Research, Westmead, New South Wales, Australia; 2 Faculty of Health and Medicine, School of Medicine, Sydney Medical School, The University of Sydney, Sydney, New South Wales, Australia; 3 Westmead Hospital, Western Sydney Local Health District, Westmead, New South Wales, Australia; 4 Department of Microbial Infection and Immunity, The Ohio State University, Columbus, Ohio, United States of America; 5 P3 Research Laboratory, College of Pharmacy, The Ohio State University, Columbus, Ohio, United States of America; 6 Department of Microbiology, The Ohio State University, Mansfield, Ohio, United States of America

## Abstract

Phage therapy is a medical form of biological control of bacterial infections, one that uses naturally occurring viruses, called bacteriophages or phages, as antibacterial agents. Pioneered over 100 years ago, phage therapy nonetheless is currently experiencing a resurgence in interest, with growing numbers of clinical case studies being published. This renewed enthusiasm is due in large part to phage therapy holding promise for providing safe and effective cures for bacterial infections that traditional antibiotics acting alone have been unable to clear. This Essay introduces basic phage biology, provides an outline of the long history of phage therapy, highlights some advantages of using phages as antibacterial agents, and provides an overview of recent phage therapy clinical successes. Although phage therapy has clear clinical potential, it faces biological, regulatory, and economic challenges to its further implementation and more mainstream acceptance.

## Abstract 2

Phage therapy employs viruses that specifically infect bacteria to combat bacterial infections, including those infections that do not respond to treatments using traditional antibiotics. Despite being developed 100 years ago, phage therapy is currently experiencing a resurgence in interest, as outlined in this Essay.

## Introduction

*“The phenomenon of bacteriophagy*, *as carried out under optimal conditions in vitro*, *is spectacular*.*”* [1]

Science’s awareness of the bacteriophage (phage) phenomenon seems to have begun by around 1898 [2–4], although the idea was not well appreciated until Felix d’Hérelle’s seminal phage paper of 1917 [5,6]. Although later it was suggested that d’Hérelle had in fact been scooped by Frederick Twort in 1915 [7,8], then, as well as today, both Twort and d’Hérelle are recognized as bacteriophage co-discoverers [9]. Prior to 1915, in addition to Gamaleya’s 1898 report, it is possible that a number of additional researchers had also discovered phage-associated phenomena, a list that notably should not include the much referenced Hankin, 1896 [10,11]. In any case, it appears to have been rapidly obvious to d’Hérelle that an entity capable of killing bacteria—at the time a key defining characteristic of phages along with their smallness and transmissibility—could have medical utility. Thus was born the concept of phage therapy [12], the treatment of bacterial infections with viruses (phage virions) to eliminate or at least reduce numbers of disease-causing bacteria [13,14], with 1921 being the year that a human phage therapy study was first published [15].

Still, phage therapy does not currently serve as a standard of care in most countries. To explore why that is so, this Essay begins by introducing basic bacteriophage biology, some of the post-1921 history of phage therapy, and also multiple advantages associated with using phages as antibacterial agents. We then turn to the growing catalog of recent clinical phage therapy successes, discussing the general nature of these studies in particular, as well as important future directions. Despite these successes, multiple obstacles to the further development, acceptance, and approval of phage therapy continue to exist, which we differentiate into biological hurdles to contrast with those we dub instead as “societal.” Overall, although we highlight the increasing potential for phages to serve as alternatives or adjuncts to antibiotic therapy for bacterial diseases, we emphasize the remaining challenges to making this promising technology more clinically available. For a complementary recent review emphasizing multiple additional aspects of phage therapy not covered here, we point the reader to Strathdee and colleagues [16].

## Bacteriophages

*“…le microbe antidysentérique est un bactériophage obligatoire*.*”* [5]

Bacteriophages, first and foremost, are viruses of Bacteria, sharing the world with viruses of Archaea and viruses of domain Eukarya. An alternative ecological categorization separates those that infect primarily “macro”-organisms (animals, plants, macrofungi, and larger multicellular algae) from those that infect microorganisms (bacteria, archaea, single-celled protists, micro-fungi, and microscopic algae) [17,18]. For the latter, virus dissemination between individual cells (e.g., between bacterial cells) and between whole organisms (also, e.g., between bacterial cells) are more or less the same thing. For the viruses of macro-organisms, especially multicellular organisms, dissemination instead is within bodies, while transmission to new individuals, including to other humans, typically is a somewhat distinct phenomenon [19]. In the following section, we review some phage therapy–relevant aspects of phage biology.

### Tailed phages

Phages recently have been differentiated into numerous taxa—particularly families and genera but also subfamily ranks—as based on genomic similarities between isolates [20]. More traditionally, at least 10 distinct phage types have been distinguished on the basis of gross virion morphologies [21,22]. These morphologies vary in terms of whether or not virions contain lipids, have tails, or contain DNA or RNA genomes, as well as whether those genomes are single-stranded or double-stranded. Smaller-genomed phages (under approximately 10 kb) generally possess single-stranded nucleic acid (DNA or RNA), middle-sized genomed phages (also RNA or DNA, but double stranded, and with genomes ranging in size from roughly 10 to 15 kb) seem to typically have virions that contain lipids, while larger-genomed phages (generally greater than 15 kb) appear to lack these lipids, have double-stranded DNA genomes, and possess tails [17,23,24]. It is tailed phages, members of virus order Caudovirales (to be replaced with class Caudoviricetes; [20]), that represent most of the phages employed in therapy.

**Fig 1:**
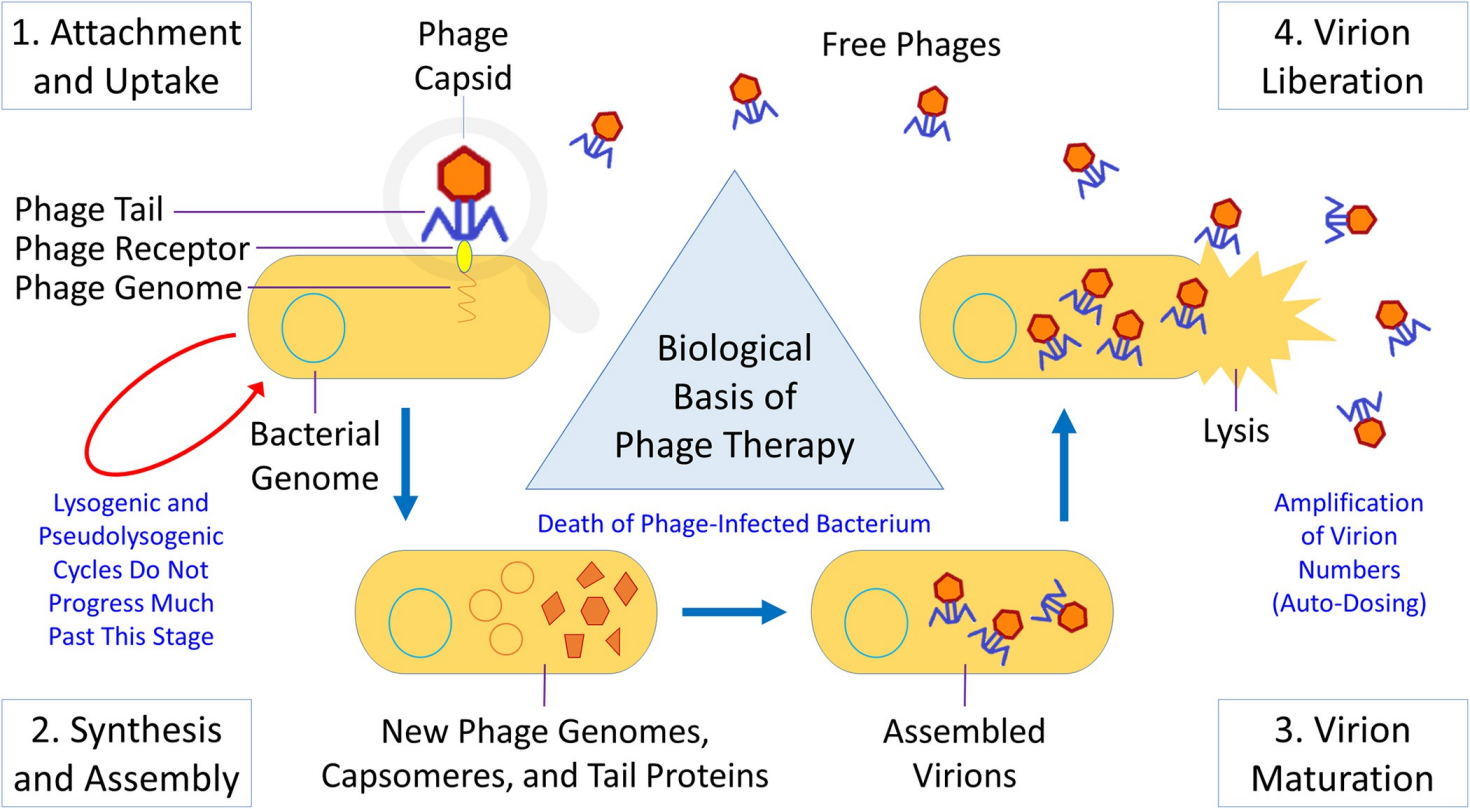
Phage lytic infection cycle. The phage infection cycle. This flows counterclockwise in the figure, starting from the upper left. (**1**) Phage attachment to receptor molecules found on bacteria [29,30] is typically described as processes of virion adsorption [31,32] with uptake involving movement of the virion genome from the phage virion into the bacterial cytoplasm. This can lead to the noted lysogenic cycles (main text) or, in some cases instead, pseudolysogeny [33–35], but as shown, particularly for virulent phages, gives rise to lytic cycles. (**2**) Synthesis is of phage-specific macromolecules including RNA, DNA, and proteins. Assembly is the process of generation of new virions from those macromolecules as resulting, ultimately, in (**3**) maturation of virions into adsorption proficient entities. (**4**) The timing of liberation of virions generally is under phage genetic control [36], though for certain types of phages (not tailed and also not shown), this release occurs chronically rather than lytically [25].

The virion-productive life cycle of all tailed phages ends in lysis of the host bacterium, initiating an extracellular search for new bacteria to infect [25] (Fig 1). This lysis breaches the bacterial cell envelope, thereby also metabolically destroying the phage-infected bacterium. Alternatively, many phages can display lysogenic cycles [26,27], which are not virion productive but during which the phage genome, now called a “prophage,” nevertheless replicates along with its bacterial host. Lysogenic cycles caused by tailed phages can eventually give rise to lytic infections, hence the term, “lysogenic,” where “lysogenic” is considered to be a property of lysogens (i.e., of bacteria harboring prophages). The phages capable of establishing lysogenic cycles should be described as temperate [28].

### Professionally lytic bacterial viruses

During lysogenic cycles, temperate phages protect their bacterial hosts from being lysed by other, related phages. This type of protection is known as superinfection immunity [37,38]. Although infection of a bacterium by a temperate phage can, and often does, result immediately in a lytic cycle [39], these phages, at least in unmodified forms [40], are not generally regarded as useful therapeutically. This typical absence from phage therapy use is due to both their lysogenic cycles (temperate phages thereby not always killing the bacteria they successfully infect) and the noted superinfection immunity (preventing other phages from killing those same bacteria). An additional factor is so-called lysogenic conversion [41], whereby many temperate phages encode bacterial virulence factors (e.g., those responsible for the intoxications associated with cholera, diphtheria, and *Escherichia coli* O157:H7) [42,43].

Even though many or even most viruses of archaea are also tailed [44], archaeal viruses generally are not described as phages [45]. Archaeal viruses have also been only minimally explored for possible therapeutic uses [46], owing at least in part to the relative dearth of archaea that are associated with disease [47–49]. Viruses of protists are also not described as phages, although these viruses too have been proposed for possible therapeutic use [49–51]. By contrast, the use of bacteriophages, and especially tailed bacteriophages, as therapeutic agents has been extensive. This Essay thus focuses on the therapeutic use of bacterial viruses that are most commonly tailed, at least ideally lack an ability to enter into lysogenic cycles (thereby being described instead as strictly lytic, obligately lytic, or virulent), and that do not encode bacterial virulence factors. Wild-type phages possessing these properties, particularly ones that are unrelated to temperate phages, can be described as “professionally lytic” [28], and those are the preferred phages for therapeutic use.

## History and advantages of phage therapy

*“Soon after Félix d’Hérelle discovered bacteriophages in association with diarrheal illnesses*, *he speculated that phages were responsible for the usual recovery from such disease through their antibacterial action in vivo*.*”* [52]

**Fig 2:**
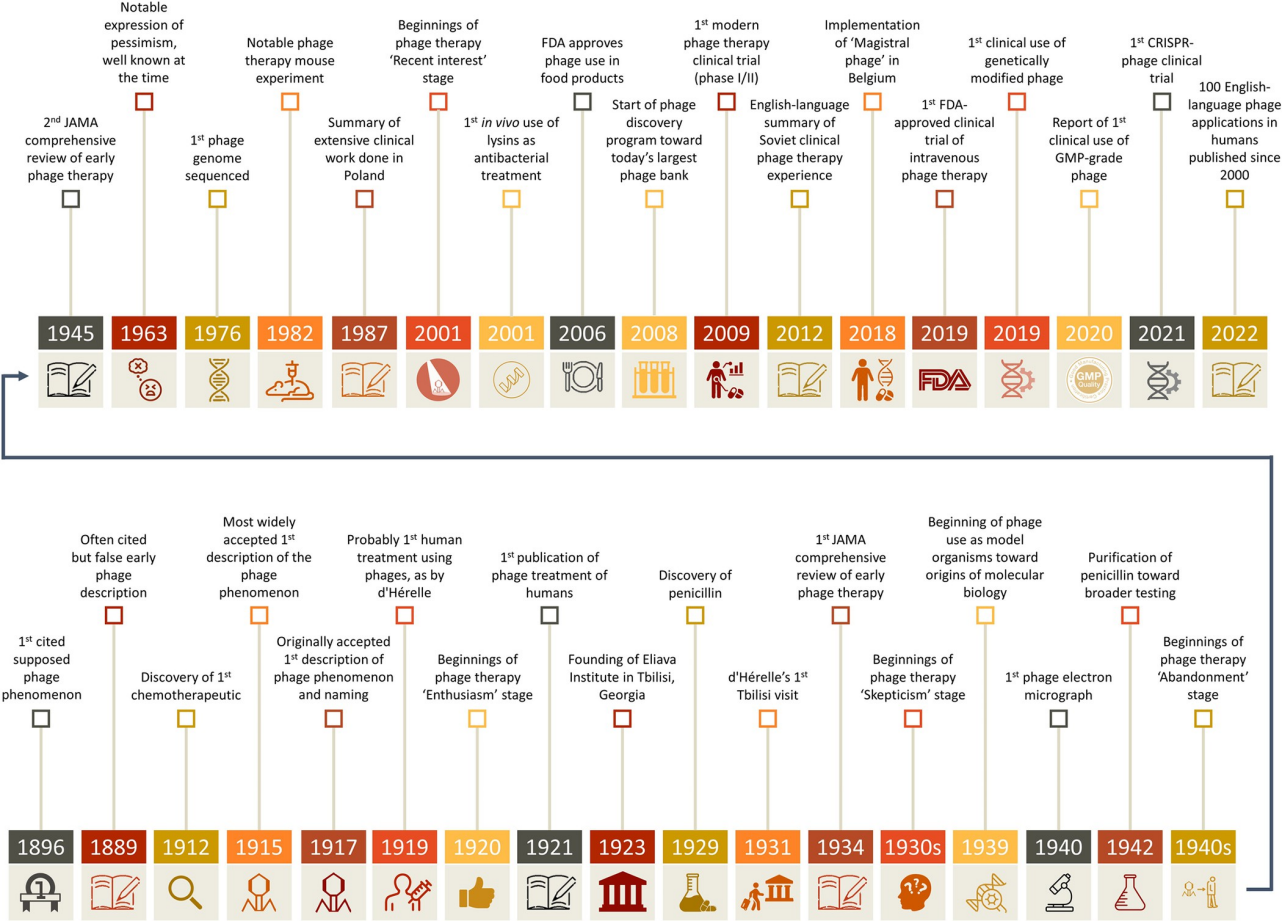
Milestones in phage science and phage therapy. Milestones in phage science and phage therapy. Abbreviations include GMP, good manufacturing practice; IV, intravenous; JAMA, Journal of the American Medical Association. Early general references include [66–68] and [1,69]. Additional references used to create the figure include from 1963 [70], 1987 [71], 2001 [72], 2008 [73], 2009 [74], 2012 [75], and 2021 [76]. Icon copyright attributions by first-use year (all as obtained via thenounproject.com and presented parenthetically; superscripts are associated country abbreviations): 1896 (Studio 365 ^TH^), 1889 (pongsakorn ^TH^), 1912 (Luiz Carvalho ^BR^), 1919 (Sergey Demushkin ^RU^), 1920 (Arafat Uddin ^BD^), 1923 (Wuppdidu ^DE^), 1929 (Irfan Setiawan ^ID^), 1931 (Adrien Coquet ^FR^), 1930s (Mourad Mokrane ^RU^), 1939 (Soremba ^DE^), 1940 (Cassandra Cappello ^CA^), 1942 (Icon Lauk ^ID^), 1940s (WEBTECHOPS LLP ^IN^), 1963 (Adrien Coquet ^FR^), 1976 (Eko Purnomo ^ID^), 1982 (One Pleasure ^ID^), 2001 (Kelsey Armstrong ^US^), 2006 (Nendra Wahyu Kuncoro ^ID^), 2008 (Creative Stall), 2009 (Kamin Ginkaew ^TH^), 2018 (Kamin Ginkaew ^TH^), 2019 (Wikimedia Commons), 2019 (Irfan Setiawan ^ID^), 2020 (Wikimedia Commons), 2021 (Irfan Setiawan ^ID^).

Historically, the translation of phage therapy from the bench to the clinic has happened at a rapid pace. This is in part because systems for testing new therapeutics were not as well developed 100 years ago as they are today. In addition, at that time, there were few alternative approaches to responding to the great deal of morbidity and mortality associated with bacterial infections [53]. It was therefore much easier to justify clinical phage therapies without prior, detailed preclinical data. Confidence in phage utility was also likely fueled by a number of apparently successful anecdotal results [54]. Furthermore, phage treatments seemed safe, resulting in minimal downsides for their clinical use, as can be particularly true in modern times with the use of purified phages for treatments [55–58]. Thus, in a pre-antibiotics world, in which standard of care for treatment of bacterial infections was highly lacking in efficacy, phages with their inherent antibacterial properties could supply a much needed hope. This does not mean that phage therapy was extremely widely practiced in the 1920s and 1930s. Still, there is ample evidence of their clinical use during this time, as a number of historical reviews have documented [12,54,59–65]. See Fig 2 for a timeline of notable phage therapy-impacting events.

### Phases of phage therapy development and enzybiotics

We can consider the historical development of phage therapy in terms of phases or periods. Especially in North America, we can distinguish these different phases into what Summers [59] described as “Enthusiasm,” the 1920s through the early 1930s [66–68]; “Skepticism,” the mid-1930s through the mid-1940s, culminating in the widespread introduction of antibiotics; “Abandonment,” the mid-1940s through the mid-1990s [77]; and then “Recent interest,” which began in the mid-1990s [64]. English language-published human studies during this revival of interest number at least 100 [78,79], and indeed we are aware of roughly 50 that have been published in just the 2020s. It is important to recognize, however, that phage therapy has been continuously practiced for almost its entire history in different locations worldwide, such as in the Soviet Union and its successor republics [75,80], but also now for many decades particularly in Poland [71,80,81].

More recently, bacteriophage-derived antibacterial enzymes, also called “enzybiotics,” have raised interest due to their typically rapid and unique modes of action along with their high specificity [82,83]. They are proteinaceous—although unlike whole phages, they are lacking in nucleic acids, thus simplifying their regulation—and, importantly, are often associated with a low propensity for bacteria to develop resistance. Two classes of phage-derived enzymes are commonly described: peptidoglycan hydrolases (also referred to as “lysins”), which degrade the bacterial cell wall, and polysaccharide depolymerases, which break down bacterial surface-associated polysaccharides. The latter’s targets can include bacterial capsules, slime layers, biofilm matrix, and lipopolysaccharide (LPS) [82,84–86]. These phage-derived enzybiotics have proven to be highly effective in animal models against gram-positive bacteria and, especially in modified forms in the case of lysins, also against gram-negative pathogens. Several clinical trials have involved lysins [82].

### Advantages of phage therapy

The inherently bactericidal nature of especially obligately lytic phages is not the only attribute that makes phages useful as antibacterial therapeutic agents [87–89]. Curtright and Abedon [90], e.g., attempted to differentiate the benefits of using phages to treat bacterial infections into ones with greater or lesser utilities. Among greater utilities, in addition to their bactericidal nature, are the potential for phages to replicate to higher doses in situ (auto-dosing), which can serve to counter processes of virion dilution and inactivation also in situ; the inherently low toxicity of professionally lytic phages, resulting in phage therapy generally being a safe approach to treating bacterial infections [56–58,91–93]; and a typical lack of cross-resistance between phages and antibiotics, although there are exceptions to the latter [94–96]. Phages with novel antibacterial activities and low toxicities also tend to be easily discovered. An additional advantage is that of limited phage impact on microbiomes, as well as the fact that reductions in bacterial functionality (antagonistic pleiotropies) are often associated with mutations to phage resistance; both of these latter utilities of phage therapy are briefly discussed in subsequent sections. Given the usefulness of phages as bacterial agents that stem from these numerous advantages, not only have phages been employed to treat bacterial infections for more than a century, but phages are increasingly being used clinically especially to treat antibiotic resistant or tolerant infections.

## Recent phage therapy accomplishments

*“The excitement about the prospects of phage therapy (PT) has been growing worldwide*, *fueled by the recent reports of its successful application in severe cases of bacterial infections*.*”* [13]

A rise in phage therapy clinical reports is noticeable starting from 2018. This represents a landmark year for the growing implementation of modern phage therapy (“recent interest”), resulting in important new English language evidence of clinical phage therapy efficacy. Numerically, while only 2 clinical reports were published in 2015, 1 in 2016, and 5 in 2017, this rises to 13 in 2018, 16 in 2019, and 11 in 2020 [78]. Our as-yet less formal counts in 2021 and 2022 further indicate that these numbers have risen again to approximately 20 each.

Many of these newer, clinical phage therapy reports have been case studies or case series conducted as compassionate treatments [91,97,98]. These thereby lack negative treatment control populations and have been deemed as possessing “low-to-moderate quality, with high risk of bias and large heterogeneity” [99]. Experts still agree, however, that these studies can support claims of phage therapy safety [56–58,91,93], with this safety having been demonstrated even in populations of critically ill patients with severe sepsis and septic shock [56,100,101]. Nevertheless, it is important to keep in mind that the primary objective of compassionate treatments is to provide therapeutic benefits to individual patients rather than to evaluate the efficacy of the treatment itself. It has been argued also that successful phage therapy case reports and series, and of course also successful clinical efficacy trials, can be viewed in a positive light supporting an observance of phage therapy anti-infection effectiveness [102].

**Fig 3:**
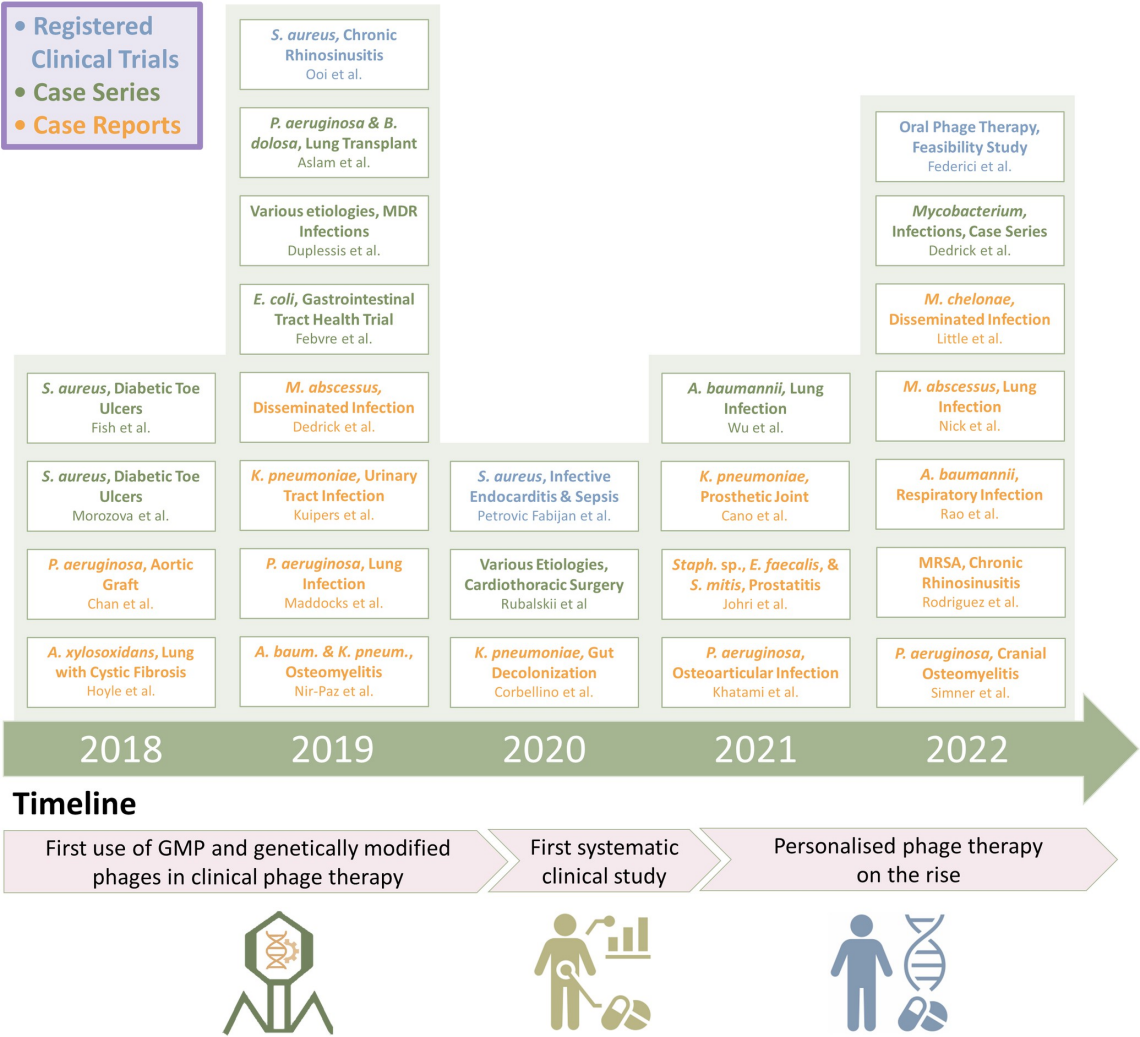
Recent clinical phage therapy accomplishments. Targets of recent phage therapy trials and case studies include *Achromobacter xylosoxidans*, *Acinetobacter baumannii*, *Enterococcus faecalis*, *Escherichia coli*, *Klebsiella pneumoniae*, *Mycobacterium abscessus*, *Mycobacteroides chelonae*, *Pseudomonas aeruginosa*, *Staphylococcus aureus*, and *Streptococcus mitis*. Abbreviations include GMP, good manufacturing practice; MDR, multidrug resistant; MRSA, methicillin-resistant *S*. *aureus*. References used to create the figure: [40,56,104–124]. See also [78] for a more complete list of modern phage therapy clinical studies showing evidence of phage-mediated efficacy. Icon attributions can be found in the legend to Fig 2, corresponding respectively to years 1915 and 2019 (in combination), 2009, and 2018.

Bearing in mind these limitations, as well as the small sample sizes of these studies—a majority are single case reports and only a few are case series—and also the nature of the studies (i.e., uncontrolled trials), we discuss below representative reports of clinical improvements during and after use of phage therapy, highlighting in Fig 3 recent studies that particularly suggest a likely clinical efficacy of phage therapy. This topic has been extensively reviewed in recent papers [16,78,91,103], but key aspects that we consider here further are the first use of genetically engineered (GMO-like) phages to treat infections, implementation of systematic analyses of phage treatments, and the rise of personalized phage therapy along with more standardized monitoring in this era of precision medicine.

### Rise of personalized phage therapy and standardized monitoring

The concept of personalized or bespoke phage therapy may be viewed as “a paradigm shift in the development and application of infectious disease therapeutics” [125]. Indeed, a majority of clinical studies have described the use of carefully selected and precisely targeted phages that have been incorporated into treatments on a patient-by-patient basis. Known as “magistral phage” [126] (or magistral phage*s*; [127]), a new, pragmatic regulatory framework is being pioneered by a multidisciplinary phage task force in Belgium (the Coordination group for Bacteriophage therapy Leuven or CBL) made up of phage scientists, pharmacists, and clinicians, and which importantly has been supported by knowledgeable authorities (e.g., the Federal Agency for Medicines and Health Products in Belgium). Collection of patient and scientific data in standardized manners, further expansion of phage banks (collections of potential therapeutic phages), and optimization of phage therapy protocols are the main concepts endorsed in the study protocol, called PHAGEFORCE [128], which many other countries in Europe and beyond have started to follow [129].

Excellent consensus documents have also recently emerged from Europe [128] and the United States of America [130] that address the need to standardize and monitor phage treatments in order to better understand phage therapy, especially within this context of personalized phage therapy. A similar level of standardization was recommended by Australian Infectious Diseases physicians in an informal survey and is reflected in the international efforts to set up a phage therapy patient database (known as “International Phagistry”) for centralized tracking of patient cases as well as standardized, unbiased reporting to ensure optimal treatments (personal communication, S. McCallin, L. Leitner, et al.). This has resulted in a more consistent approach to treatment and monitoring of phage therapy, acknowledging differences in targeted etiologies and routes of phage administration, but focusing on process, safety, and monitoring [129].

While important hurdles remain to be addressed, the current use of phage therapy and still somewhat informal but improving evidence of its efficacy suggest that phage therapy likely will be introduced to the mainstream especially as a personalized medicine. Employing the abovementioned standardized and multidisciplinary approaches will help identify and address major scientific and treatment hurdles for furthering the acceptance of phage therapy in the context of personalized medicine, but also help to improve the design of randomized controlled trials.

### Genetic engineering of therapeutic phages

Engineering can enhance the therapeutic potential of bacteriophages [76,131–133]. This can be achieved directly through alteration of host range (e.g., via homologous recombination or mutagenesis of tail fiber genes) [134–136], modification of the phage infection (e.g., via deletion or deactivation of genes required for lysogenic cycles) [137,138], or modification of the phage capsid (e.g., via selection of phages capable of remaining in the circulatory system for longer times) [139,140]. Phages can also be modified to enhance the antibacterial activity of conventional antibiotics, such as by engineering into them an ability to produce quorum sensing–interfering factors [141] or the noted biofilm-matrix degrading enzymes [142]. Since the inception of molecular biology, these and a number of other methods have been developed to engineer phages, approaches that have been recently reviewed in a phage therapy context [16].

A breakthrough study by Dedrick and colleagues [40], in 2019, described the first published clinical application of genetically modified phages to treat a clinically disseminated infection caused by the notoriously antibiotic-resistant *Mycobacterium abscessus*. For this case study, 1 naturally obligately lytic phage (phage Muddy) and 2 temperate phages (BP and ZoeJ) were identified that could effectively kill the clinical isolate, starting from a library of over 1,800 phages. To generate a therapeutic cocktail, the authors employed a Bacteriophage Recombineering of Electroporated DNA (BRED) technique [143] to remove lysogenization modules from the 2 temperate phages. The subsequent phage therapy course of 7 months, which included both topical and intravenous administration of the adapted phage cocktail, was reported to be well tolerated and resulted in significant clinical improvement. A further narrative of the case can be found here: [144]. This study was then followed by a case series involving the phage treatment of 20 additional patients, also including use of genetically engineered phages, further demonstrating both their safety and effectiveness as antimycobacterial agents [122].

It thus has only been in the past few years that genetically engineered phages have been employed clinically to treat bacterial infections, targeting only a single bacterial genus (*Mycobacterium*) and used for compassionate care (also see discussion in [76]). Carefully designed and controlled clinical trials are therefore still needed to give definitive answers on the potential for phage genetic engineering to improve therapies, as is also the case with traditional phage therapy involving natural phages. Phage engineering promises to generate therapeutics with unique properties, however, thereby offering alternative treatment approaches in the management of difficult-to-treat bacterial infections along with a potential for stronger patent protection (below).

### Systematic analyses of clinical studies

While the safety of phage therapy has been demonstrated in more than 50 studies conducted since 2000 [58], systematic analyses of clinical and microbiological phage treatments are relatively scarce. Most published clinical phage treatments have involved patients treated on compassionate grounds, which restricts the collection and systematic analysis of scientific data. Moreover, the limited sizes and heterogeneity of reported studies (e.g., diverse pathologies, use of different single phages or instead phage cocktails, and different administrations protocols) makes it almost impossible to conduct robust meta-analyses. Nonetheless, 2 recent systematic analyses have been conducted reporting favorable clinical and microbiological outcomes attributed to adjunctive phage therapy in at least 60% of treated patients [56,122].

In 2020, a study conducted as a single-arm, noncomparative trial explored the safety and tolerability of phage therapy in 13 severely ill patients with *S*. *aureus* bacteremias, including infective endocarditis [56]. A systematic analysis was conducted of bacterial and phage kinetics in the blood as well as inflammatory responses. In addition, the microbiological outcomes were assessed via comparative whole-genome sequencing analysis of bacterial isolates collected before introduction of phage therapy, as well as any isolates retrieved during phage therapy, to ensure that no phage resistance developed in vivo. To our knowledge, this study was the first of its kind to provide a comprehensive and multimodal assessment (including clinical, microbiological, and immunological assessments) of critically ill patients undergoing phage treatment.

In 2022, a case series involving 20 patients with non-tuberculosis *Mycobacterium* infections reported safety and tolerability of phage therapy, using the noted natural and genetically modified phages [122]. The authors conducted microbiological and immunological monitoring of patients, and, although the study was conducted on compassionate basis, it provided valuable insight that should serve as a fundament for design of future randomized controlled trials in the field.

## Continuing challenges for phage therapy

*“Notwithstanding the extensive need*, *interest*, *experience and reported successes of phage therapy*, *typical western approaches to biomedical research and implementation are poorly adapted to motivate*, *regulate or assess such nonstandard approaches to antibacterial therapy*.*”* [145]

**Fig 4:**
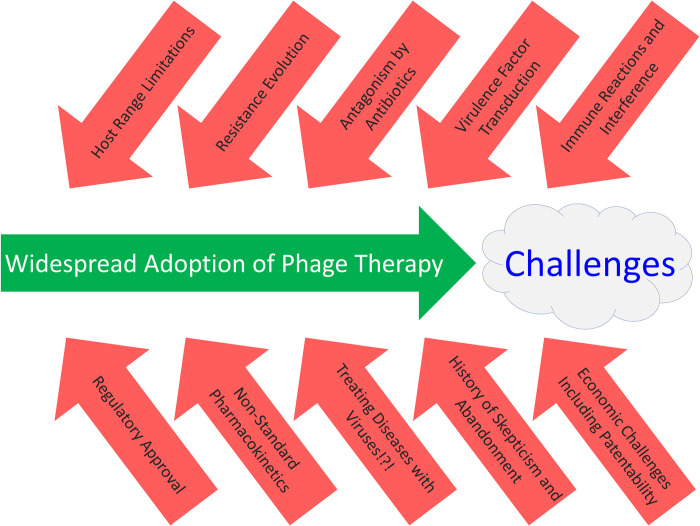
Challenges to more widespread adoption of phage therapy. At the top are issues that are more biological in their character, whereas at the bottom are issues that are more societally imposed impediments to greater phage therapy implementation. Not shown but associated with the latter are difficulties that physicians can face in simply obtaining treatment phages in countries where phage therapy is not already a standard of care.

Phages not only offer numerous advantages as antibacterial therapies but also, despite their ongoing and increasing use for treating bacterial infections for which antibiotics are not or no longer useful, present a number of challenges to their increased application. These challenges include issues of limitations to phage host ranges and thereby spectra of activity, the potential for development of bacterial resistance to phages, possible negative impacts of antibiotics on phage functionality, treatment phage-mediated transduction of bacterial DNA, interactions with the immune system, regulatory issues, unusual pharmacology, insufficient awareness of phages as therapeutic antibacterial agents, and the potential for phage therapy skepticism (Fig 4). We differentiate these concerns into biological or instead societal challenges. Additional complications include those related to manufacturing and storage of therapeutic phages, but we refer readers instead to [146,147] for discussion of those issues.

### Biological challenges

Although obligately lytic phages are inherently bactericidal, that does not mean that their existence as potential antibacterial agents is without limitations. Many of these issues, along with phage-induced bacterial lysis, are aspects of the pharmacodynamics of phage therapy, particularly treatment-phage impact on bacteria but also their effect on treated bodies [148]. Though presented as ongoing challenges to the implementation of phage therapy, these biological issues are not necessarily inevitably negatively impactful on the potential for successful treatments, particularly given prior knowledge of their existence.

#### Host range limitations

The host ranges of phages tend to be relatively narrow, typically consisting of only a subset of strains within a single bacterial species [149,150]. Indeed, an often stated benefit of phage therapy is the resulting somewhat narrow spectrum of activity, particularly compared to most commercially available antibiotics. This is because treatment phages have a reduced potential to give rise to adverse effects due to an only minimal negative impact on beneficial, nontarget commensal bacteria [111,151–154]. Disadvantages, nevertheless, can come with this limited phage spectrum of activity, and these are at least 2-fold. First, it makes it more challenging to treat bacterial infections empirically when using phages compared with broader-spectrum antibiotics. Second, it means that greater numbers of unique phage products need to be developed overall, i.e., at a minimum one for every bacterial species to be targeted.

A partial answer at least to the first concern (limitations on empirical treatment), but in some cases to the second as well (at least 1 formulation per bacterial species targeted), is the development of what are known as phage cocktails [155–157]. Cocktails are preparations that possess multiple phage types, ideally including phages with different host ranges. If the species of a to-be-treated bacterial infection has been determined, then it is possible to use cocktails, which specifically target that bacterial species. Alternatively, phage cocktails that target specific disease types rather than just individual bacterial species can be used and have been particularly in countries of the former Soviet Union, such as those for wound-associated infections (a pyophage cocktail) or gastrointestinal infections (an intestiphage cocktail) [61,158–160]. These tend to consist of multiple phages each targeting an individual bacterial species, and this is rather than individual phages instead targeting multiple bacterial species. Rather than mixing different wild-type phages to generate phage cocktails, it is possible instead to modify the host ranges of existing phage isolates either by phage evolution [161], also known as phage training [162], or via genetic engineering.

#### Bacterial resistance to phages

It is the nature of genetic entities to evolve, if they can, in response to environmental conditions. This is abundantly true in response to chemotherapies, whether what is being treated is abnormal cell growth within bodies or bacterial populations being exposed to antibiotics. The same is true for bacteria and phages [163]. We can differentiate resistance to phages or chemotherapies into that which is acquired and that which is inherent in a given bacterial species. Acquired resistance can, in turn, be differentiated into that which comes to stably dominate bacterial population prior to exposure to an antagonist and that which instead comes to prominence only once exposure has begun, with the latter of interest here especially when resulting from treatment-mediated selective pressures [148]. To both antibiotics and phages, we can distinguish also between resistance acquired horizontally, especially in the course of bacterial acquisition of mobile genetic elements [164,165], and that which is acquired mutationally, i.e., as resulting in modification of bacterial molecules required by phages to successfully infect [166]. Furthermore, and also true for antibiotics, we can split bacterial mechanisms that interfere with therapies into those that arise in otherwise-sensitive bacteria only phenotypically (forms of tolerance) and those that arise genetically (resistance) [167].

Mutations to phage resistance, in particular, can result in antagonistic pleiotropies (trade-offs) [167]. These, generally speaking, are phenotypes that are associated with individual alleles that, on the one hand, provide benefits to their carrier, such as that of phage resistance by bacteria, but, on the other hand, result in a cost to their carriers. Such bacterial functional trade-offs may be particularly prevalent following phage therapies because phages can benefit by targeting bacterial molecules that are difficult for bacteria to do without. Examples of negative phenotypes associated with phage-selected antagonistic pleiotropies can include reduced bacterial growth rates, decreased bacterial virulence, or increased bacterial sensitivity to antibiotics [168–172]. Phage-resistant bacterial pathogens may, as a consequence, become diminished in their ability to continue to cause infections. See, too, the related concept of “Directing phage-resistance evolution” [173].

Limitations to phage host ranges are a corollary of phage resistance, and both can be addressed by the use of phage cocktails. This usually is accomplished by combining phages with complementary host ranges to increase empirical coverage for a single targeted bacterial species. Application of novel combinations of antibiotics can also be justified toward increasing empirical coverage [174]. The generally lower toxicities of phages, however, should enable the routine use of novel phage combinations to treat patients, not just when addressing medical crises, i.e., while employing untested combinations of antibiotics to treat patients should be attempted only under more desperate circumstances, given that untried combinations of drugs can give rise to unexpected side effects, there exist few similar barriers to employing new combinations of especially professionally lytic phages in phage cocktails. Indeed, there exists little expectation that novel phage–phage interactions will result in substantial or even necessarily any anti-patient toxicities.

To employ phage cocktails to limit bacterial capacities to evolve resistance, it is generally necessary to include within cocktails not just multiple phage types possessing complementary host ranges but also more than 1 phage host range type that is able to infect the targeted bacterial strain, and for which bacterial mutation to cross-resistance to both (or more) cotargeting phages is of low probability. In other words, to reduce the potential for bacteria to evolve resistance to phages by employing phage cocktails, it is necessary that cocktails possess a sufficient “depth” of antibacterial activity [155], with a depth of 1 being a single phage isolate within a cocktail able to kill a targeted bacterium, a depth of 2 being 2 phage isolates within a cocktail able to independently target, thereby with minimal cross resistance, that same bacterium, and so on. This is either in addition to or instead of phage cocktails possessing substantial overall breadth of host ranges (broader spectrum of activity), i.e., as is needed to better assure at least the initial success of empirical treatments rather than necessarily also inhibition of bacterial evolution of phage resistance [155].

#### Interactions with antibiotics

As antibiotics represent the standard of care for treatment of bacterial infections, it is inevitable that phage therapies will often be undertaken as antibiotic cotherapies [78]. As is the case for antimicrobial treatments generally, we can expect the interactions between phages and antibiotics to range from antagonistic to additive to synergistic. Antibiotic antagonism of phages especially may be expected, as antibiotics often interfere with bacterial processes required for phage infection success [175–179]. In addition, the fact that antibiotics can reduce bacterial numbers on their own will contribute to a reduced potential for phages to amplify themselves in situ [180]. It is unknown, however, to what extent this in vitro–demonstrated antagonism actually impacts clinical treatments.

Alternatively, combining antibiotic and phage treatments can increase overall effectiveness. In order of increasing effectiveness, this can variously be described as involving facilitating, additive, or synergistic interactions [181]. These are, respectively, some but less than additive increases in the elimination of targeted bacteria relative to each agent acting alone (facilitative), each agent impacting bacteria independently of the other’s actions (additive), and greater than additive bacteria killing (synergism). Synergistic interactions between phages and antibiotics, especially at subinhibitory antibiotic concentrations, can also result in what is described as a phage-antibiotic synergy (PAS) [32,147,179,182,183]. With PAS, the presence of such low antibiotic doses actually enhances certain aspects of phage infection, contrasting the noted potential for higher, inhibitory doses of antibiotics to antagonistically interfere with phage infections.

We can also differentiate whether phages in combination with antibiotics are more effective at simply clearing susceptible bacteria in the near term or, instead, are more effective at interfering with the evolution of phage or antibiotic resistance over longer time frames. Near-term improvements could be associated with PAS but can also result from simply additive interactions, as well as the noted facilitation. Longer term, the typical lack of cross-resistance to phages and antibiotics should increase the number of bacterial mutations required to achieve full resistance to a combined treatment [184,185]. This is the usual goal of combination treatments [174,186–189] including the employing of phage cocktails possessing greater-than-1 depths of activity. Alternatively, acquisition of phage resistance can result in bacteria becoming susceptible once more to antibiotic effects (resensitization) [106,170,190,191]. Notwithstanding both the inevitability and potential utilities of combining phages with antibiotics, the resulting pharmacodynamics, particularly when treating using bacteria-inhibiting doses of specific antibiotics along with specific phages, remain substantially understudied.

#### Transduction

Transduction is phage virion-mediated movement of non-phage DNA between bacteria [192]. It is possibly problematic during phage treatments owing to the potential for phages to transport bacterial virulence factor genes between different bacterial strains. This includes transporting antibiotic-resistance genes as well as bacterial pathogenicity islands and bacterial plasmids [193–196]. Transduction, however, is a distinct phenomenon from lysogenic conversion [197], since transduction involves accidental and short-term virion packaging of bacterial DNA, compared with long-term carriage of bacteria-like genes by wild-type temperate phages (lysogenic conversion). There are 3 basic contexts in the course of phage therapy where transduction could be problematic: movement of DNA from the bacteria used to propagate therapeutic phages in vitro for subsequent therapeutic use; movement of DNA from nontarget bacteria to therapeutically targeted bacteria; and movement of DNA from targeted bacteria to nontargeted bacteria. The first of these can be avoided by employing bacteria for phage propagation that lack relevant virulence factor genes. The second and third can be reduced in likelihood by employing therapeutic phages that possess sufficiently narrow host ranges.

It is also possible to simply avoid using phages that are capable of transducing bacterial DNA, i.e., by not using so-called “transducing phages.” The latter include temperate phages, with their ability to move small numbers of bacterial genes in what is known as specialized transduction (though, again, this is not equivalent to lysogenic conversion). Of probably greater relevance to phage therapy is avoidance of phages that package their DNA using *pac* sites and headful genome packaging, both in combination with phages not substantially degrading the DNA of their host bacterium in the course of lytic cycles [198]. Phages possessing these latter properties can act as generalized transducing phages, i.e., phages that are able to solely package bacterial DNA up to approximately a phage genome size in length and then transfer that DNA to new bacteria.

Of the biological challenges facing phage therapy, the potential for transduction nevertheless is often considered a lesser concern [199]. This is because transduction is a natural process that happens independently of any therapeutic introduction of phages and is often associated with temperate phages that tend to be avoided for phage therapy independently of any transducing phage status. Also, an otherwise untreated bacterial infection would be of much greater immediate negative impact. Thus, as noted by Ry Young [200], “Unless you’re completely compulsive, it doesn’t make a whole lot of sense to me to worry about transduction.”

#### Immunology

In clinical settings, phage–bacteria dynamics develop in conjunction with pressure from the mammalian host immune system, and this has often been stated as a substantial concern regarding the clinical implementation of phage therapy. In particular, there are concerns that a patient’s immune status will influence the effectiveness of phage therapies, but also that phage–immune system interactions might be harmful to patients. Alternatively is the concept of “immunophage synergy,” as presented by Roach and colleagues [168], where both neutrophils and phages were necessary for resolution of acute pulmonary infection in mice. Phages also have immunomodulatory properties, impacting the function of major populations of immune cells that contribute to both innate and adaptive responses [201]. This includes, among other responses, cytokine production (different from LPS-induced cytokine profiles) [202], phagocytosis [203], the respiratory burst of phagocytic cells [204], and production of antibodies against non-phage antigens [201]. Phages are also a part of the normal human microbiome [205], with some phage virions adhering to mucosal surfaces and thereby potentially serving as a non-host–delivered layer of immunity [206–208]. Thus, the role of the immune system in phage therapy appears to be multilayered and, indeed, in practice, is likely to be less of a challenge to phage therapy success but instead an important contributor to that success.

Phages can be found, in particular, in the human gastrointestinal tract, including, e.g., those infecting Firmicutes, Bacteroidetes, Proteobacteria, and Actinobacteria [207,209,210]. Phages thus represent core components of stable gut microbiomes, and they are consequently immunologically well tolerated by the body. Consistently, in a recent case series conducted at the Phage Therapy Unit at the Hirszfeld Institute in Poland, a majority of patients did not show noticeably higher levels of anti-phage antibodies in their sera during oral and local phage cocktail treatment of methicillin-resistant *S*. *aureus* infections [211]. Even if the humoral immune response was greater, anti-phage antibody production did not seem to give rise to unsatisfactory clinical results. The intensity of the anti-phage humoral response, however, may vary depending on phage type, its inherent immunogenicity, and the purity of a phage preparation [12,212]. Phage dosage, timing of treatments, routes of administration, and the immune status of a patient also may contribute to the impact of anti-phage humoral immunity on phage therapy success [201,213,214]. Nevertheless, it is important to be aware that anti-phage antibody production does not necessarily mean phage inactivation [201] as it also depends on antibody titers and specificity. One can also consider selection or engineering of phages that will be more resistant to antibody-related inactivation [76,202].

An additional issue during phage therapy is that treatment of gram-negative pathogen infections with high phage dosages may lead to a synchronized lysis of large numbers of bacterial hosts along with concomitant release of endotoxin. This triggers inflammation, typically via Toll-like receptor 4 pathways, as well as fever and may cause local pain [120]. Nevertheless, this LPS release does not necessarily exceed the amounts generated in the course of antibiotic treatments [215], and a septic shock syndrome following phage treatment of these pathogens has not been reported. Indeed, it is often observed in studies that phage treatments are associated with no more than minor adverse effects, if any at all. Thus, concerns that have been expressed over the potential for the immune system to interfere with phage therapy efficacy appear to have been somewhat exaggerated.

### Societal challenges

Widespread implementation of nonstandard treatments by physicians requires not just evidence of efficacy but also regulatory approval, successful marketing, and a willingness of those involved to undertake such treatments. In this section, we consider these additional, societal challenges to broader adoption of phage therapy, especially by Western medicine.

#### Regulatory issues

In the former Soviet Union, phages were mass produced for therapy and have long been available, even over the counter [61,216], with the first phage therapy trials there dating back to the 1920s [217]. Elsewhere, phage therapy has been available almost only for “compassionate use” [91,97,98] (i.e., when need is great and all else has failed) and particularly for treatment of pan-drug-resistant infections or otherwise following antibiotic treatment failures (i.e., with phages serving as salvage therapies). Despite the recent progress in many aspects of the development of phage therapy, the introduction of phages into the clinic still faces major obstacles [147,218,219], and this is particularly from unresolved regulatory questions [220].

To move forward, either current regulations will need substantial modification or new legislation will need to be proposed to cover aspects of phage therapy that differ from those of traditional antibiotics. Regulations, in particular, need to accommodate phage genetic malleability (their potential to evolve), their narrow host range, and their unusual pharmacological properties. Both US and European regulatory authorities at least agree, however, that therapeutic phages should be classified as biological therapies [221], requiring compliance with fairly well-defined legislative, manufacturing, and production frameworks. Many experts, including regulatory authorities such as the US Food and Drug Administration (FDA), also agree that evidence of phage therapy efficacy from controlled clinical trials, of which there so far have been only a very limited number, are essential to accelerate development of regulatory frameworks.

Because existing regulations have been developed for industry-scale production of medicines, they are less well suited to the more personalized approaches of phage therapy [147]. Insufficient flexibility and exemptions within these frameworks, e.g., to allow for the introduction or substitution of new phages into approved preparations in response to resistance development, has had a chilling effect on market uptake and the otherwise widespread application of phages in therapies [222]. In Europe, authorities are making an effort to streamline the use of personalized phage therapies, with many countries following Belgium’s pioneering approach of regulated magistral phage preparations tailored in a context of compassionate care for specific infection and patient cases [147,223]. Well prior to those efforts, the Hirszfeld Institute in Wrocław, Poland, has had a many decades-long history of involvement in personalized phage therapy as too has been the case in and around the Eliava Institute in Tbilisi, Georgia [80,81].

#### Unique pharmacology

An important component of successful regulatory approval of drugs is a robust characterization of their associated pharmacology. Study of the pharmacology of phages has been somewhat neglected, however, owing to how long historically phages have been used to treat bacterial infections, which largely has predated the development of modern pharmacological study, and also, arguably, due to the relative safety of phage use as antibacterial agents, which have made concerns over potential toxicities somewhat moot [148]. In addition, study of the pharmacology of antibacterial agents has largely been developed from a perspective of use of antibiotics. Thus, the challenge of the uniqueness of phage use as antibacterial agents can be viewed as somewhat of a societal construct, i.e., had antibacterial pharmacology as a science been developed based primarily on the properties of phages rather than those of antibiotics, then it presumably would be antibiotic pharmacology that is “unique,” relative to phages, rather than that of phages relative to antibiotics. This section thus provides an overview of phage therapy pharmacology and does so particularly from a perspective of how phage therapy pharmacology differs from that of antibiotics. Overall, the challenge in this case is to provide both regulatory agencies and physicians with detailed analyses of phage pharmacokinetics (PK) during treatments.

Pharmacology can be considered, in particular, along the traditional divisions of PK and pharmacodynamics (PD), and this is true for phage therapy pharmacology as well [147,224,225]. PK refers to mechanisms that influence drug distribution to target tissues, including to the vicinity of targeted bacteria, particularly to result in drug concentrations that are adequate to achieve effects [226]. Those effects are described by the PD component of pharmacology, with the most relevant PD effect being antibacterial activity. It is phage therapy PK that can differ somewhat from that of other pharmaceuticals, while primary phage therapy PD are conceptually similar to other antibacterial agents in terms of the effect of killing bacteria. We therefore focus here especially on the unusual PK of phage therapy.

A drug’s PK can be broadly defined by a handful of key identifiers. These include area under the curve (in situ concentration as a function of time) divided by minimum inhibitory concentration (AUC/MIC), maximum drug in situ concentration also divided by MIC (C_max_/MIC), and the fraction of time between dosings over which in situ drug concentrations exceed MIC (abbreviated as *t*>MIC). These key PK identifiers, however, have not been as well defined for phages as they have been for antibiotics. This is, at least in part, because although multiple groups have used checkerboard-type assays to describe phage MICs [191,227–229], there, nonetheless, is no standard method for defining phage MICs and nor has there been robust in vivo validation [55].

Difficulties in defining phage therapy PK as well as MICs stem largely from the potential for phages to proliferate during treatments. Compounding this complication, the extent of this in situ phage proliferation will tend to vary with phage properties (particularly as impacting the productivity of their infections), the properties of targeted bacteria, and the properties of the treated infection itself (perfusions, immune factors, adjunct antibiotic effects, etc.). Another confounding variable is the noted PAS [147,179,182,183], where especially sub-MIC concentrations of certain antibiotics can boost phage production. Moreover, titers of phage particles relative to concentrations of targeted bacterial cells may greatly impact PK parameters, particularly with these phage concentrations varying nonlinearly over time in response to that ratio [230]. Also relevant is that achieving initial phage concentrations in a range of 10^8^ to 10^9^ per ml (thereby presumably requiring less in situ phage proliferation) may be useful toward achieving treatment success [55,56,58,225] and that phages can take multiple hours to reach maximum concentrations, such as in the blood, with that timing varying with different routes of administration.

Especially when phage proliferation is less influential, then AUC and thereby phage therapy PK should be more similar to that of traditional pharmaceuticals. This should be seen particularly with so-called passive treatments [224,231,232] where dosed phage concentrations alone—like dosed antibiotic concentrations alone—should be high enough to kill a majority of targeted bacteria, assuming adequate bioavailability and distribution. An additional issue, relevant perhaps especially to the continuation of bacterial killing once numbers of phage proliferation-supporting bacteria have substantially declined in concentrations, is that phage counts, like antibiotic concentrations, will tend to decrease over hours following dosing [233–236]. The rates of phage elimination of dosed phages from the body (as another important PK identifier, defined in terms of half-life or t_1/2_) also may vary with routes of phage administration [226,237].

The stage that an infection is treated is another significant factor affecting phage therapy PK. Biofilm-resident bacteria, which are often found later during chronic bacterial infections [54], can be in less active growth states and vary in their virulence [238], potentially affecting their tolerance of phage treatments [167]. In addition, bacterial stationary phases can often be antagonistic to productive phage lytic infections [34,239–241] as too can inhibitory concentrations of antibiotics. It also can be difficult to extrapolate from the in vitro activity of any antibacterial agent, including bacteriophages, to clinical outcomes, since host factors including infection sites and types are both major treatment–outcome determinants but will tend to differ from patient to patient. Thus, phages can fail to resolve an infection caused by a pathogen even if those phages appear to be powerfully active in vitro. Phage therapies, nonetheless, may be more promising than antibiotics in some cases in the treatment of biofilms [54,106,242,243].

While phage therapy PK can be somewhat more difficult to define than those of antibiotics, the in situ phage proliferation underlying much of that difficulty, i.e., phage auto-dosing, is likely also a particular advantage of phage therapy. Nevertheless, to achieve consistently effective phage treatments, routes and dosages of phage administration must be evaluated and standardized to each specific phage–host-infectious disease combination [226,244,245]. Given the diversity of phages that are or could be available for phage therapy use, however, achieving such evaluation and standardization on a phage-by-phage basis could represent a daunting impediment to robust phage therapy clinical implementation.

#### Societal awareness of phage therapy

Trust in phages and phage therapy remains strong in former Soviet Union republics, particularly Georgia and Russia [62,216]. In the Western world, in contrast, appreciation of phage therapy has only gained momentum in recent years, with a growing number of cases highlighting efficacy in treating various multidrug-resistant infections. These range from lung infections in patients with cystic fibrosis (CF) [40] to treatment of urinary tract infections [246] to resolution of the most severe infections [247], including phage use as treatments for gram-positive sepsis and septic shock [56]. Although most of this evidence is anecdotal, reports of phage therapy accomplishments have led to increased media coverage of phage therapy, fueling interest in this new therapeutic alternative among the public. Though sadly not representing a phage therapy success, the story of Mallory Smith—a young patient with CF who died of a pan-drug-resistant lung infection prior to the initiation of phage treatment [248]—in particular, has raised awareness of the importance of phage therapy as a potential treatment option in this vulnerable population.

There has been a corresponding increase in funding for phage research and initiation of a number of controlled clinical trials in the field [78,93,103,130,144,147,249]. With cautious optimism, pioneers of modern phage therapy are establishing national and international initiatives where basic scientists and clinicians can work jointly to professionalize phage therapy (Table 1) by using well-defined and standardized treatment approaches [129], thereby adding to the work of somewhat more established phage therapy units and centers (particularly those of Georgia and Poland). The ultimate aim of such initiatives is to better align this therapeutic option with the priorities of main funding and regulatory bodies, clinics and pharmacopeia, and, finally, of patients themselves. Alternatively, for a list of phage therapy as well as simply phage-based commercial entities, see Phage Companies.

**Table 1:** Modern phage therapy centers, groups, and initiatives.

Name	Country/Region	Reference
Phage Australia	Australia	[250]
Monash Phage Foundry* (Monash University)	Australia	[251]
START Phage WA*	Australia	[252]
The Adelaide Phage Therapy Centre	Australia	[253]
Coordination group for Bacteriophage therapy Leuven (CBL)	Belgium	[254]
Queen Astrid Military Hospital	Belgium	[255]
Farncombe Phage (McMaster University)	Canada	[256]
Phages for Human Applications Group Europe (P.H.A.G.E.)	Europe	[257]
“Phage Therapy Unit in Finland” (University of Helsinki)	Finland	[258]
Reference Center for Complex Osteo-Articular Infections	France	[259]
Eliava Consortium	Georgia	[260]
National Center for Phage Therapy	Germany	[261]
Vitalis Phage Therapy	India	[262]
The Israeli Phage Therapy Bank	Israel	[263]
Phage Therapy Unit of the Medical Centre of the Institute of Immunology and Experimental Therapy PAS	Poland	[264]
phageSuisse	Switzerland	[265]
Centre for Phage Research (University of Leicester)	United Kingdom	[266]
UK Phage Therapy	United Kingdom	[267]
Center for Innovative Phage Applications and Therapeutics (University of California San Diego)	United States	[268]
Center for Phage Biology and Therapy at Yale	United States	[269]
Center for Phage Technology (Texas A&M University)	United States	[270]
Tailored Antibacterials and Innovative Laboratories for Phage (Φ) Research (Baylor University)	United States	[271]

*Partnering with the national Phage Australia.

Physicians appear to be playing less of a role in driving the resurgence of interest in phage therapy. A survey from 2019 conducted in the largest Belgian hospital and biggest phage therapy center in Western Europe (Queen Astrid Military Hospital) indicated that more than 70% of phage therapy requests came from patients themselves or their family members and only one-third or so from treating physicians [272]. This was attributed to a lack of inclusion of phage therapy in medical school curricula or, as one clinician explained [273], “Colleagues don’t know phages rather than are opposed to them,” and phage therapy otherwise is often regarded by physicians as an “inaccessible possibility.” Thus, awareness of phages as potential treatments of bacterial infections certainly is growing but is not nearly universal.

#### Phage therapy skepticism

A somewhat unique challenge to the use of phages as antibacterial agents is a combination of their long history and insufficiently well-documented efficacy. The former allowed for the initial “enthusiasm” for phage use. Particularly, their ability to target bacteria but not our own tissues (selective toxicity) was at the time (in the 1920s and 1930s) without peer among readily available medicaments. This, however, also provided little incentive to study the clinical use of phages rigorously. In addition, many successful anecdotal case studies were poorly documented, and it is thought that many treatment failures were a consequence of poorly formulated or applied therapies. Phage therapy’s early challenges to rigorously establish itself also stemmed from a dearth of well-controlled clinical trials (although with some exceptions; [102,217]) in combination with the potential for many of the bacterial infections being treated to spontaneously resolve [66–68]; notably, today, we have a similar issue as many phage-treated infections are also being treated with antibiotics, which, in principle, can lead to infection resolution that is independent of phage action [78]. In any case, insufficiencies in scientific rigor appears to have allowed for a growth in “skepticism” over the therapeutic potential of phage therapy, a skepticism that lead to a Western “abandonment” that lasted for roughly 50 years. See chapter 3 of Kuchment [274] for a narration of these events.

Together, these issues seem to have resulted in long-standing cultural impediments to phage therapy implementation [275]. Skepticism and, ultimately, Western abandonment of phage therapy are not thought to have been driven primarily by public perception, however, but rather by the actions of physicians or, rather, by their inaction. Importantly, it is not as though phage therapy was ever implemented widely in most countries even when enthusiasm was strongest, thus allowing for small shifts in what relatively few physicians were practicing to drive substantial declines in phage therapeutic use. The same cannot be said for the use of antibiotics, which were widely adopted once they had become available in sufficient quantities [276]. The decline in enthusiasm for phage therapy was not the case everywhere, and this, at least in part, was because the use of antibiotics was not as well implemented everywhere, e.g., in the former Soviet Union [102]. The abandonment of phages by Western medicine was driven, however, not just by the ubiquity of antibiotics but also by the fact that broadly targeted antibiotics are simply easier to use than narrowly specific phages as antibacterial treatments. Today, we can also add economic impediments as obstacles to broader phage therapy acceptance by Western medicine.

#### Patentability

Further complicating the broader implementation of phage therapy are economic uncertainties associated with phage therapy development. While biotech companies often succeed in translating basic research into profitable clinical applications [219], investment into phage therapy nonetheless raises many concerns, not least of which is the limited patentability of phages, along with unmodified enzybiotics [277], as “natural phenomena” or “product[s] of nature” [278]. US patents covering the use of natural phages in therapy nevertheless have been granted [279], many of them claiming that specific phage cocktails are essential to reduce the risk of resistance development by targeted bacteria [280]. Such patents are considered fragile, however, and thereby will not necessarily provide robust commercial protection [218]. Genetically engineered phages with enhanced antibacterial activity by contrast may be more easily protected [221] and thereby could serve as a safer focus for government and private investors [281–283].

Naturally occurring, i.e., not genetically engineered phages, by contrast are overwhelmingly what have been used in the development as well as clinical implementation of phage therapies. But without robust patentability, there is less financial incentive to invest in the kind of vigorous research necessary to overcome phage therapy skepticism, particularly including the funding of clinical trials, and also toward financing phage therapy commercial development more broadly. The fragile patentability of naturally occurring phages for phage therapy thus may represent the greatest societal challenge to phage therapy and, indeed, challenge to phage therapy generally in those many countries where phages are not yet regulatorily approved as antibacterial treatments. We return to yet further considerations of phage therapy economic issues in the following, final section of this Essay.

## Translating phage therapy to the clinic

*"Although more translational research is needed before the clinical implementation of phage therapy is feasible*, *phages may be pivotal in safeguarding the overall health of humans in the near future*.*"* [284]

There is a wealth of preclinical as well as more basic science-derived data supporting the potential for phage therapy use clinically. At the other extreme, various phage therapy centers and initiatives (Table 1), companies, and established research groups are actively involved in developing and testing phage collections and treating people. Occupying something of a middle ground are physicians who do not, on their own, have access to phages, or at least easy access, but who are able to link up with centers, companies, or research groups to obtain those phages. Initiatives such as Phage Directory facilitate connections between phage suppliers, such as from academic research laboratories, with possible phage clinical users, i.e., doctors [285].

Overall, then, there are 4 general routes to human phage therapy. The first is administration without clinical supervision, which is possible in places where phages are available over the counter, particularly Georgia and Russia [286]. The second is use by physicians or equivalent caregivers in locations where phages have been approved for use clinically (e.g., also Georgia and Russia). Here, phage therapy was regulatorily approved and translated into the clinic many decades ago. The need for practicing phage therapists to demonstrate efficacy in formal and very expensive clinical trials, therefore, is less pressing. The third approach, and one that currently receives the most attention, is within the context of dire need, i.e., involving compassionate use as well as, typically, personalized medicine. This approach lends itself less well to providing proof of efficacy as controls are difficult to establish and combination of phage treatments with use of standard-of-care antibiotics is usual [78]. In this context, phage therapy typically is sought for the toughest of bacterial infection cases as salvage therapies. This potential for phages to successfully treat bacterial infections for which antibiotics have been less effective, however, provides a possible niche for both phage therapy use and phage therapy testing.

Lastly are clinical trials, which are necessarily limited in scope and expensive to run, but which are essential for the explicit proof of phage safety and efficacy that regulators, prescribers, and consumers need. To date, a handful of phage therapy clinical trials have been published in English language journals (for English language access instead to especially the Russian and Georgian literatures, see [75]). Though modern phage therapy clinical trials started out with much promise, especially with the Phase I/II trial reported by Wright and colleagues [74], at best subsequent trials seem to have met with only mixed results [287,288] and otherwise face many challenges [55], though this may change as increased public funding for phage therapy clinical has become available [144]. In particular, phages are most likely to be tested under circumstances where antibiotics have already been attempted as the first option but otherwise may be found to be superior to antibiotic treatments in only a limited number of circumstances. Nevertheless, there is great potential to choose phages as alternatives to unacceptably toxic antibiotics [289] or to reduce problematic antibiotic impacts on microbiomes [111,151–154].

A final and nontrivial problem is the reduced commercial enthusiasm to bring new antibacterial agents of any kind from the laboratory to the clinic. There is a general expectation that antibacterial agents will not only be used for only short periods of time (i.e., from the point of infection presentation to the point of infection cure) but will be relatively inexpensive during that ideally somewhat brief use [290]. Together, these economic obstacles represent significant disincentives not just to phage therapy translation to the clinic but also to the introduction of new antibiotics more generally. Added to this, but more specific to phages, is the noted issue of the uncertainty of intellectual property security of naturally occurring biological agents. However, to the extent that it may be proven in the course of modern clinical trials that phage treatments are able to cure bacterial infections when conventional treatments have failed and/or that we become serious as a society, or at least as subsets of society, to protect our microbiomes, then clinical phage therapies may yet rise again, not just in a few select locales but also around the world. Indeed, as a final word, we both suggest and agree that phage therapy might be viewed as a third major intervention for treating infectious diseases after vaccines and antibiotics [250] and are buoyed by phage therapy’s recent clinical successes (Fig 3) and growth in use despite numerous existing challenges.

## Abbreviations

AUC: area under the curve
BRED: Bacteriophage Recombineering of Electroporated DNA
CF: cystic fibrosis
FDA: Food and Drug Administration
LPS: lipopolysaccharide
MIC: minimum inhibitory concentration
PAS: phage-antibiotic synergy
PD: pharmacodynamics
PK: pharmacokinetics
